# “This is hard to cope with”: the lived experience and coping strategies adopted amongst Australian women with pelvic girdle pain in pregnancy

**DOI:** 10.1186/s12884-022-04426-3

**Published:** 2022-02-02

**Authors:** Dragana Ceprnja, Lucinda Chipchase, Pranee Liamputtong, Amitabh Gupta

**Affiliations:** 1 Western Sydney University, Sydney, New South Wales Australia; 2 Physiotherapy Department, Westmead Hospital, Sydney, Australia; 3 Flinders University, Adelaide, South Australia Australia; 4 College of Health Sciences, VinUniversity, Hanoi, Vietnam

**Keywords:** Pregnancy, Women, Pelvic girdle pain, Qualitative, Interview, Experience, Coping

## Abstract

**Background:**

Women with pregnancy-related pelvic girdle pain (PPGP) report diminished ability to perform physical activities and experience higher rates of mood disorders, such as anxiety and depression, than pregnant women without PPGP. Despite these physical and psychological impacts, little is known about the lived experiences of PPGP amongst Australian women and the ways in which they cope. Situated within biographical disruption and social support theories, this study sought to gain a conceptual understanding of the experience and impact of PPGP on daily life, and how women cope with this condition during pregnancy.

**Methods:**

A qualitative research design, situated within a phenomenological framework, using individual, semi-structured interviews consisting of open-ended questions was used with a flexible and responsive approach. Purposive sampling of pregnant women attending a single hospital included 20 participants between 14 and 38 weeks gestation, classified with PPGP as per recommended guidelines, with a mean (SD) age of 31.37 (4.16) years. Thematic analysis was performed where interview data was transcribed, coded, grouped into meaningful categories and then constructed into broad themes.

**Results:**

Three themes were identified: 1. a transformed biography; 2. coping strategies; and 3. what women want. The pain experienced created a dramatic change in women’s lives, making the pregnancy difficult to endure. Women utilised social support, such as family, to help them cope with pain, and a self-care approach to maintain a positive mindset and reduce stress. Although a few women received support from healthcare professionals, many reported a lack information on PPGP and limited societal recognition of the condition. Women wanted early education, personalised information and prompt referral to help them cope with PPGP.

**Conclusions:**

Findings from this study highlighted the complexity of living with PPGP as women attempted to deal with the unexpected impact on daily life by seeking support from partners and families, while also struggling with societal expectations. Although women with PPGP used a number of coping strategies, they sought greater support from healthcare professionals to effectively manage PPGP. These findings have important implications for the provision of health care to women living with PPGP.

**Trial registration:**

Australian New Zealand Clinical Trials Registry: ACTRN12618001423202.

## Background

Pregnancy tends to be perceived as a joyous time in women’s lives. Society portrays a picture of bliss and happiness during pregnancy, often highlighting the positive moments such as feeling the baby’s first movements “kicking” and the pregnancy “glow”. However, there are unpleasant physical symptoms associated with pregnancy, including nausea, fatigue, heartburn and musculoskeletal pain which do not attract the same amount of media attention [1]. Of these physical symptoms, pregnancy-related pelvic girdle pain (PPGP) is a common pain reported universally during pregnancy [2]. Pain is located within the pelvic area between the posterior iliac crest and gluteal folds, with or without leg pain [3]. Although prevalence rates globally have been reported to range from 7 [4] to 84% [5], a prevalence rate of 44% was determined recently in an Australian population [6].

Women with PPGP report a significant reduction in their ability to perform daily activities, such as standing, walking and turning in bed, and face major challenges in their role as a mother, undertaking household chores and work-related tasks [2, 3, 7–12]. However, PPGP does not only cause physical impacts. Women with PPGP experience higher rates of mood disorders, such as anxiety and depression, suffer sleep disturbances, and have a lower quality of life than their pregnant counterparts who do not experience pain [4, 7, 10, 13].

Despite the physical and psychological impact on daily life, little is known about the lived experiences of PPGP and the ways women cope with it. Coping has been defined as any cognitive or behavioural attempt, successful or unsuccessful, to manage conditions that are perceived as difficult or induce stress [14]. In order to understand the lived experience and coping strategies adopted by women, a qualitative approach is essential to allow for a deeper exploration and understanding from the perspective of women who live with PPGP [15]. A recently published systematic review of eight studies describing the experiences of women found that pain had a major impact on women’s lives and families [7]. However, of the eight studies, only three included women classified with PPGP using recommended guidelines [9, 11, 12]. These three studies, all conducted in Sweden, reported that women struggled with daily life, avoided movement at home and work, and redistributed household tasks to partners, parents and other relatives [7, 9, 11, 12]. Although these studies reveal valuable information about the Swedish experience of coping with PPGP, there has been limited investigation into PPGP using qualitative methodology in Australian women, and the strategies Australian women use to deal with pain remain unknown.

Previous studies have identified that women value support of health care professionals and the interventions that they provide, as well as information about the condition [7, 13]. However, it has been suggested that current health care services do not meet the needs of women struggling to cope with PPGP [2, 8, 11, 12, 16]. In an Australian study, only 25% of women with lumbopelvic pain received any type of treatment for their condition, despite telling their health care provider about their pain [8]. The reasons for low treatment rates are unknown, but a lack of knowledge about PPGP has been suggested to result in poor management and outcomes [7, 16]. In order to deliver healthcare that truly meets women’s needs, efforts must be made to explore what existing and /or additional supports women feel may assist them to cope with PPGP. This has not been extensively examined previously worldwide.

Thus, this paper aimed to explore the lived experience of Australian women with PPGP including the impacts on daily life, the coping strategies they adopted, and what additional supports they may require to help them better cope with PPGP. Knowledge gained from this qualitative enquiry will inform healthcare professionals of the care needs of the women they seek to support during pregnancy.

### Theoretical frameworks

This study is situated within biographical disruption theory [17] and social support theory [18]. These theoretical lenses allow for examination and interpretation of the responses from women which are complex, real-world problems.

Biographical disruption theory, coined by British sociologist Michael Bury (1982), provides insight into how people respond and adapt to illness, suggesting that it is a “major kind of disruptive illness” [17]. The theory highlights that biographical disruption does not result from a disorder, but from the ways that disorder impinges on one’s physical ability to engage with daily life [19]. Suffering from pain during pregnancy can bring disorder to a woman’s life and impact on her ability to perform activities of daily living. Bury (1982) also suggested that attention needs to be provided to the actions that individuals actively seek to counter the impact on their lives [17]. Therefore, the strategies cultivated by women to help them cope with PPGP were explored.

Social support has been theorised as needing to be mobilized in response to the changes in life and social relationships [17]. In social support theory, the effects of stressful life events on health are reduced through the supportive actions of others or the belief that support is available [18]. Supportive actions may enhance coping and are categorised into four behaviours including emotional, instrumental, informational and appraisal. Thus, the social support theory provided real-world knowledge about what women need to be able to cope with PPGP.

## Methods

### Design and setting

A qualitative research approach provided a rich description of the lived experience of women living with PPGP, allowing for women to describe their experiences of how PPGP impacted on daily life and the strategies used for coping with PPGP [15, 20]. Qualitative research was deemed vital as little is known about the lived experience of women with PPGP in Australia. This study was situated within a phenomenological framework in order to understand what the first-hand experience means for women with PPGP [15]. In line with the phenomenological framework, the semi-structured individual interviewing method was used to collect the data in this study. The method allowed the participants to articulate their lived experiences in detail [15].

This study was conducted at Westmead Hospital in Sydney, Australia, from November 2019 to February 2021. Westmead Hospital is a large teaching and tertiary referral government funded hospital in an urban centre with over 5200 births recorded annually [21]. The hospital has a catchment area that includes women from a diverse range of socio-economic and ethno-cultural backgrounds, educational levels and working status [6, 21].

### Participants

Women attending Westmead Hospital for antenatal care were provided with written and verbal information about the aims and methods of the study by the first author (DC) and assured of their confidentiality and privacy being maintained. Potential volunteer participants were also informed that they could withdraw from the study at any time without their ante-natal or health care being affected.

Women were included if they were over 18 years of age, between 14 and 38 weeks gestation, classified with PPGP and had a sufficient command of the English language to be able to be able to provide written and informed consent and complete the interview. All participants were classified as having PPGP according to recommended guidelines, which included a physical examination [3]. Participants were excluded if they self-reported any medical or obstetric complication(s) that may have affected pregnancy including pre-eclampsia, eclampsia, serious intellectual or psychiatric impairment, systemic disease(s), or recent spinal fracture, trauma or surgery.

Stratified purposive sampling was used to ensure that the sample was representative of women with PPGP who attended Westmead Hospital such that participants were intentionally selected according to the needs of the study [15, 22–24]. Attempts were made to include women with a culturally and linguistically diverse background who were able to speak English. This ensured that a range of voices were heard and provided opportunities for women to be able to share their individual experiences who may otherwise not be included in this type of research [15]. In addition, a broad sample of women was sought, including those who had a partner and those who did not, women of differing paid employment status, and there were no restrictions placed on parity.

### Sample size

Using the qualitative approach, the sample size required was determined when saturation of themes was achieved such that the collection of new data did not add any further information on the aims of the study [15]. Studies situated within the phenomenological methodology have suggested that this requires between 5 and 25 participants [25]. In this study, it was planned that at least 25 women would be interviewed to ensure richness of the data with a broad and diverse sample. However, no new information emerged after the tenth participant interview. After discussion amongst the research team, the decision was made to continue to collect data in order to further probe and explore themes to be certain that that no further information became available with more interviews and to allow for deeper understating of issues raised by women. Previous research has reported that “code saturation” may indicate when researchers have “heard it all”, but “meaning saturation” is needed to “understand it all” [26]. Thus, our final sample included 20 women with PPGP in order to develop a richly textured understanding of the lived experience of women with PPGP. Ethical approval was granted by the Westmead Hospital (2019/ETH02528) and Western Sydney University (H12532) human research ethics committees.

### Procedure

The first author (DC) contacted each participant by telephone to offer a face-to-face interview or the option to complete a written diary to explore their lived experiences. The written diary method offered was originally planned to allow the participants to document their experiences in a diary at a time convenient for them, and described in the published protocol [27]. However, all participants chose to attend a face-to-face interview. The interview was scheduled at a mutually convenient date and time at Westmead Hospital with each participant receiving reimbursement for the cost of transport or parking to the value of AUD$20.00.

On attendance, each participant completed a written questionnaire to determine anthropomorphic characteristics (age, height, body mass) and information about their current pregnancy, including weeks of gestation, parity, pregnancy type (singleton, multiples), and current pain level using a visual analogue scale [28]. Further information that was collected included country of birth, self-identified ethnicity, marital status, current physical activity level, and work status.

### Interviews

An interview guide consisting of open-ended questions was used with a flexible and responsive approach to ensure that the same range of topics were discussed with each participant (Table 1). Key questions were followed by further prompts as relevant to the individual interviewee. Although, follow up questions differed slightly between participants, using an interview guide ensured the key topics were covered in all interviews [15]. The interview guide was developed by the researchers and the questions aimed to determine the lived experience of having PPGP. Women were questioned about how PPGP impacted their daily life, what changes to the performance of daily activities they had made, and how they felt they coped, including what strategies they used to help them cope with PPGP. Women were also asked about what other support(s) or information should be provided to help them be able to better cope with PPGP.

**Table 1:** Interview Guide Questions

Questions	Prompts
How do you see your pelvic girdle pain?	Tell me about your pelvic girdle pain. What is it like? Can you explain to me what having pelvic girdle pain in pregnancy means to you?
How do you feel about having pelvic girdle pain in your pregnancy?	Tell me about how it makes you feel. What feelings does the pain bring out?
How does having pelvic girdle pain in pregnancy affect your daily life?	Does the pain make certain activities harder? How does having pelvic girdle pain in pregnancy affect you at home? With family? At work? In social situations?
What changes to the performance of daily activities have you made as a result of having pelvic girdle pain in pregnancy?	How do you do things differently? Do you change positions more often? Why do you have to do this? How does this help? Do you rest more? How does this help? Tell me about what activities you have stopped doing or do less of because of having the pain?
In your view, do you feel you can cope with having pelvic girdle pain in pregnancy?	Do you feel you are able to cope? How do you see this? What does coping mean to you?
What do you do to help you cope better?	Do you ask others for help at home or at work? Do you outsource tasks, such as cleaning, online shopping? Have you changed tasks at work? Do you limit your activity? Do you receive any treatment? Use a pelvic belt?
What supports have you received to help you cope with pelvic girdle pain?	From where? Any supports from midwives, physiotherapists, doctors or other health care professionals? Tell me about the supports. What about any information from ante-natal education programs, brochures, fact sheets, online resources? Other people, such as relatives, friends, and work colleagues?
What other things do you think would help you cope with pelvic girdle pain better?	What about more information? Earlier information? Tell me about what would you like from health care professionals? From family? Friends? Work colleagues? What about referrals for treatment? Medication? Braces? Complimentary/alternate treatments, such as acupuncture?

The interviews were digitally recorded and all participants were assigned a coded number and pseudonym. The interviews ranged between 45 to 60 min in duration. The interviewer made written notes during and after each interview which included observations, thoughts and ideas about the participants’ responses [29]. These notes were then discussed amongst the research team to determine whether changes to the interview guide were needed to further explore the responses and themes offered by women. For example, based on a response by a participant early in the study, a question was added to the interview guide asking women about their views on judgement by others whilst experiencing pain during pregnancy. Similarly, women were questioned as to whether they felt they needed to plan ahead as a coping strategy when trying to deal with PPGP.

The interviews were transcribed verbatim by the first author (DC) within a week of completion and the transcript was then provided to participants. Member checking ensured that participants were able to review their responses and make edits if they felt more information was needed or to reword text they were not comfortable being included [15].

### Data analysis

Thematic analysis was conducted whereby each recorded interview was listened to several times to make sense of the data and the interview as a whole [15, 30]. A realist approach was adopted to explore women’s experiences, meaning data were accepted at face value, participant’s responses were taken to be a true reflection of their experiences, and quotes were interpreted literally [30]. Open coding was conducted by naming sections of the participants’ narratives in the text [31]. There was regular discussion between all authors to ensure thorough and consistent coding patterns of the data. To enhance trustworthiness, codes were then grouped to form meaningful categories as agreed upon by all authors. Further, analysis of three transcripts was conducted by two authors (DC and AG) independently to see if similar categories emerged. The next step was to construct broader themes from the categories. Two authors met initially and discussed themes (DC and PL) with further discussion and comparison amongst all authors, moving back and forth between text and categories to enrich data credibility. As a consequence, some of the original categories were combined, while others were separated until the final framework of broad themes was established [32]. An audit trail was also adopted to ensure the rigour of the study, providing detailed clarification of the reasons for analytical and theoretical choices [15]. In accordance with accepted qualitative methods, data collection, transcription and analysis was carried out concurrently to be able to determine when saturation was reached [33].

The consolidated criteria for reporting qualitative research (COREQ) checklist was followed [34]. A published protocol details the rationale and proposed methodological approach of the design for this study [27]. Changes to the methods were needed due to the COVID-19 pandemic. As a consequence, the study deviated from the published protocol by only utilising individual interviews as physical distancing measures, in line with government guidelines, restricted the use of focus groups.

## Results

Twenty women with a mean (standard deviation (SD)) age of 31(4.3) years between 22 and 39 weeks’ gestation participated in this study. All women were married or in a de-facto relationship. Women self-identified their ethnicity as being Australian (40%), from the subcontinent (25%), Asian (15%), Middle Eastern (10%) or European (10%). All women reported singleton pregnancies, and more than half the women (55%) had not had a previous pregnancy over 24 weeks gestation. All women reported completing high school with 65% having tertiary qualifications. Of the 20 women, 15 reported being in paid employment. Low levels of physical activity were reported amongst the sample, with 40% of women seldom participating in sport or exercise (less than once per week) and a further 30% reporting participation only once per week. The mean (SD) pain reported was 68 (23) mm on a visual analogue scale.

Three broad themes were identified and incorporated a number of subthemes following thematic analysis of the interviews (Table 2). Verbatim quotes from the interviews are used to support these themes using pseudonyms to protect the identity of participants.

**Table 2:** Broad themes and subthemes

Broad themes	Subthemes
A transformed biography	An unexpected reality Impact on the day to day Perceived judgement
Coping strategies	Social support Self-care Care from health care professionals
What women want	Acknowledgement from others More information

### A transformed biography

The pain experienced by these participants created a dramatic change in their lives. Women’s narratives suggested that they underwent an unexpected and transformed biography that had a significant impact on their day to day activities, with an additional sense of being judged by society.

#### An unexpected reality

Women found the pain due to PPGP to be a surprise and were unprepared for the impact it had on their lives, being different to what they anticipated and resulting in a view that they had not been realistic in their expectations of pregnancy. The pain made the pregnancy harder, and women felt the pain robbed them of enjoyment during this time of their lives, highlighted by the following:

*“It is so different to what I thought it would be like. I thought I would feel good and be really healthy. But this is so different to what I thought, because of the pain I don’t feel like I look the same. I feel my body is not mine” (Tamara).*

Women expressed a range of feelings regarding PPGP including annoyance and frustration, with many believing it was unfair and questioning “why me?”. Women described feeling unhappy, sad and depressed in response to the pain, with some being concerned about how much they would be able to tolerate. As a consequence of the pain being severe, two women decided they did not want further pregnancies: *“With this pregnancy very, very bad experience. No more pregnancy after this I tell husband no more. That’s it” (Leah).*

Women were perturbed about the changes to their mood, describing themselves as irritable and bad-tempered. Unexpected sentiments of guilt were also raised by some women, with respect to how they were feeling towards their unborn child and with some feeling they had let their existing family down. Often, women questioned their roles as a mother and wife, and were dismayed to feel that they were not able to do a good job in either role.

*“I am feeling guilty about how I am feeling about this baby. Thinking … maybe better to not have baby. As a mother, there’s always guilt. For my son now we are not doing any activities, which is not fair for him. Imagine as a mum how you are feeling, your child is just watching tv and you are just lying down. I wanted to help but I can’t do it” (Leah)*

#### Impact on the day to day

All women expressed that pain limited their physical activity with a decreased tolerance for walking, standing, bending and lifting. The pain also interfered with their ability to perform activities of daily living such as cooking, cleaning, shopping and driving. Movement was slower and it took a longer time to complete tasks: *“Makes things harder and I have to allow more time to do things. It’s harder to do the housework, that needs a bit of effort” (Rose).*

Some women discussed the impact of pain on their social life. They did not do as much because of the pain due to physical demands, such as standing, when socialising and psychologically not feeling in the mood to socialise. Women could not maintain their normal exercise levels, and this change in physical activity was difficult to accept.

*“I’m used to being really active and fit, and now with the pain it limits what I can do so I’m not feeling like myself, you know not as fit and active as I am usually like. Getting used to this change is hard for me to take” (Peta)*

#### Perceived judgement

Women did not want to complain for fear of being viewed as a “whinger”, with many feeling that they would be judged by others if they revealed that they were struggling during their pregnancy or showed any weakness, such as having pain. Hence, they did not want to reveal the truth of their experience, instead wanting to show the world they were having a “good” pregnancy*.* Despite self-identifying as being strong and resilient, women often expressed that they were at the mercy of society’s judgement because of their gender.

*“I’m a strong person, not really bothered by social media and stuff normally, but I feel a pressure in pregnancy to show a certain side to the world … and it can be challenging to deal with. There is judgement of women everywhere and this is just one form of it I guess” (Sofia)*

Women also went to great lengths to avoid appearing ungrateful for being pregnant. Women expressed that telling others about their pain may be judged as taking pregnancy for granted. Some spoke of how fortunate they were to be pregnant in the context of other women experiencing fertility struggles, yet found the experience of pain to be no less difficult to deal with.

*“I don’t want to appear ungrateful, because like I am lucky to have two and now be having three on the way, but … I guess it was just hard and unexpected” (Kitty)*

### Coping strategies

Despite the disruption to their biography, women attempted to find ways to cope with the pain and several coping mechanisms were described.

#### Social support

Social support was essential to the women. They expressed a reliance on others for assistance to perform tasks and chores that they would have normally performed themselves. Their partners were their biggest support, helping with household activities such as cooking, cleaning and shopping. Women valued the moral support and felt fortunate for the tangible assistance provided by partners and family*: “My husband and family help out all the time, I can’t really ask for more” (Carly).*

Some women were reluctant to ask for help to avoid being a burden to others. Additionally, women were less likely to receive help if their partner was busy at work, whilst others felt they did not receive sufficient support if their family was not near. A couple of women reported a lack of social support with their needs being dismissed by family, friends and colleagues*.*

*“No empathy from others, people just expect you to deal with it. I think it is more about people just dismissing the pain and not paying any attention to it. I think for me it is just ignored really” (Sara)*

#### Self-care

Women attempted to stay positive with an optimistic outlook where possible. They tried to not push themselves too hard, and advocated for a self-care approach to coping with the focus on their emotional, physical and spiritual wellbeing. They spoke of the importance of being kind to themselves by resting when needed and not feeling guilty about taking time out for themselves. In the face of a lack of options offered to them, women felt the need to practice self-preservation.

*“I think you need to take care of yourself and try and keep the pain to a level that’s not too bad. There’s not a lot else you can do, but you can look after yourself” (Rose)*

Focusing inwards, women used affirmative self-talk to stay positive through their pregnancy and tried not to let trivial or insignificant things bother them.

*“I think it is important to stay calm and have a positive outlook so that the little things don’t get to you” (Leanne)*

By organising themselves and planning ahead, women felt more in control of being able to cope with tasks that needed to be performed. Although this reduced spontaneity, women placed value on making choices that suited them. The demands of the household were difficult to meet, so women prioritised what needed to be done and set themselves lower standards in order to reduce some of the stress on themselves*: “I am trying not to put pressure on myself for things to be perfect” (Jennifer).*

Women looked forward to a time when they no longer had pain, highlighting that a hopeful mindset helped them maintain perspective and endure the pain.

*“I think I cope because I know there is an end point in sight, like it’s not like I’m going to be pregnant forever so I know there is an end point coming. I think this helps me cope. I just focus on getting through this pregnancy, you know like ... just get through to the other side and then it will be better*” *(Kitty)*

#### Care from health care professionals

Women sought care from midwives, doctors and physiotherapists to help manage their pain. Specifically, women reported care from physiotherapists including massage, prescription of exercise and stretches, provision of pelvic belts, and education. Women were very appreciative of the practical support afforded by physiotherapy. Most often, the midwife made the referral to physiotherapy and women were grateful for their efforts towards organising care. However, some women described having to take the initiative themselves by actively seeking referrals to physiotherapy.

*“I had to ask three times to get a referral to physiotherapy. They didn’t refer me straight away. I asked twice, thrice and then they said we will send you to physio” (Emma).*

Many suggested they would give other women suffering from PPGP advice to seek help early as they had found it easier to deal with the pain.

*“I would say go and get help early. Ask to see the physio and don’t leave until they give you the referral. See the physio and do the stretches. The stretches are really the best thing. And you don’t know what you should do without the physio” (Eloise)*

Women largely attempted to avoid taking medication, as they were unsure about the safety of medication during pregnancy or the effects it may have on their baby. For many, medication was a last resort if there were no other options.

### What women want

Although women with PPGP reported using a number of coping strategies, they sought more help from health care services. Having their pain validated was important to women, together with a desire for personalised information which was meaningful to them.

#### Acknowledgement by others

Support and acknowledgement of the pain was considered integral to women’s experiences when coping with pain. Women felt cared for if they had a good relationship with midwives, doctors and physiotherapists. They felt reassured when examined by someone they considered an expert and greatly appreciated the recognition, support and care provided.

*“Yes, the physio saw me and gave me information, had a look at my back and pelvis. I am not saying you come out of the pain like magic or something, but at least you feel supported” (Cassie)*

In contrast, women who did not receive an assessment were not satisfied with their health care and did not feel comforted: *“No one checked where the pain was or looked at it. How can I feel reassured that it is nothing serious when no one even looked at it?” (Sofia).*

Some women felt they were brushed off by health care workers, saying their “concerns were dismissed”, the pain was “normalised” and they were left to “fend for themselves”. Some went even further stating that they felt the focus was entirely on the baby in pregnancy, with a lack of attention towards the mother.

*“I think some recognition of the pain and some empathy would have been nice. Like as soon as you mention you have pain to the midwife or doctor, it’s just shut down. Like the conversation ends. No one follows up, they don’t ask you anything more about it. I think there is no focus on the mother in pregnancy, it’s all around the baby” (Sara)*

#### More information

A small number of women received information to meet their healthcare needs. Those who received information found it increased their knowledge about PPGP, assisting them to manage the pain. However, the majority of women reported a dearth of information regarding PPGP, with some suggesting they had not received any information at all so they were “in the dark” about PPGP. Many commented that receiving information earlier during their pregnancy about PPGP in the form of written material and brochures may have been helpful. However, some indicated a preference for education and advice to be tailored to their individual needs, including the opportunity to ask questions.

*“I gather that everyone is busy in the hospital, but it doesn’t take much to ask about the pain and then give information to the person so they can know about it and ask questions. I guess that would help make us feel empowered” (Sara)*

Other women reported seeking information on the internet due to the limited material from health care services or providers. A few women found information from the internet helpful, yet most suggested that it was hard to find credible information from Australian sources. Women spoke of having to “trawl through a lot of information”, and being unsure of what was good information, preferring hospital sources of information for their trustworthiness and credibility.

*“You are not sure who you can trust on the internet and what information is true. I much prefer to ask at the hospital … you know … ask people who I can trust” (Georgia)*

## Discussion

The findings of this study revealed that PPGP transformed the biography of the women as it was an unexpected reality of pregnancy. The experience of pain made the pregnancy difficult, both psychologically and physically. Women reported a number of strategies that helped them cope including having social support and accessing health care services. These findings are supported by previous studies conducted in Sweden and England [9, 11–13], with the further finding that women expressed self-care as a strategy to coping with pain during pregnancy.

As theorised by the biographical disruption theory [17], the impacts of pain were both unexpected and unwanted, and caused a change in the physical ability of women to engage with daily life. The pain also had negative effects on women’s body perception, self-identity and psychological wellbeing, which all combined to further disrupt their biography [17]. As Bury posited, in response to the changes in life and social relationships, resources must be mobilized [17]. In this study, resources in the form of support from others, including partners, family members and health care professionals, assisted women to cope with the interruption caused by PPGP. In particular, help from partners and families with household chores was paramount.

Women with PPGP placed a high value on credible information and empathetic care from healthcare services. This is consistent with previous studies of people in pain who have a desire for personalised information [35] and in studies of pregnant women with gestational diabetes who expressed a need for clear information from trustworthy sources [36]. Women also appreciated treatment by physiotherapists, preferring an active approach to management rather than a pharmacological one. Thus, the provision of information early in pregnancy and timely referrals to relevant healthcare professionals for personalised management may lessen the impact of biographical disruption to women’s lives.

Women’s ability to cope with PPGP was hampered by a perception of judgement from others. Women commented that they felt like failures if they were not seen as having a “good” pregnancy. They were also keen to not “complain” or “whinge” for fear of being labelled as weak or demanding. In the face of this perception of judgement, many women were reluctant to reveal the truth of their experiences, hiding their pain from others, suffering in silence and not seeking support to cope with pain. While not directly captured in this study, the perception of this external judgement has been identified previously to impact on women’s moods [11]. Judgement of women is not a new phenomenon, but our work in pregnancy identifies the need to address community attitudes enabling women to voice their concerns without fear of repercussions and seek required support.

Expanding on the concept of self-care as a coping strategy in Australian women, self-care has been defined as positive actions and the adoption of behaviours to minimise the impact of illness [37]. To combat the negative emotional effects associated with pain, such as low mood and anxiety, in our study, women adopted self-care approaches such as a positive mindset, and prioritised their needs to reduce pressure on themselves and minimise stress. Women may assume this approach because they perceive there to be limited options to help them cope, or to exert their own independence and take responsibility towards self-management of pain, perhaps motivated by a maternal desire to be able to nurture their growing baby.

Australian women discussed their experience of pain as creating some strain on their family, although it was not to the degree described by Swedish women in Elden et al. (2013) who spoke of “breaking points” in relationships [11]. The reasons for the differences in this finding may be due to ethno-cultural differences in the sample populations or the practice of self-care emphasized by the women in the current study. Possibly, Australian women may be reducing the stress placed on family relationships by utilising self-care approaches to maximise coping. Further investigation of the outcomes afforded by self-care is clearly warranted [37]. Nonetheless, health services may need to explore opportunities to promote self-care behaviours to women with PPGP, which may include provision of education, skills training and e-health options, to improve patient outcomes.

### Strength and limitations

The methodology for sampling ensured a large cohort of women from various backgrounds who provided rich, authentic experiences. The catchment of the hospital in Western Sydney includes women from a wide range of ethno-cultural backgrounds with over 50% of residents born overseas [6, 21]. This diversity is common to many urban cities in Australia and globally, thus providing a voice to women who may otherwise not be heard to tell their stories of pain during pregnancy. The thorough process of probing the emerging narratives provided opportunities for more women to share their lived experiences regarding themes, leading to increased confidence with saturation of information in this study. Women expressed gratitude for having the opportunity to share their experiences, provide opinions and articulate concerns, which was empowering and helped validate their pain, common to previous qualitative studies investigating PPGP [9, 11–13].

There are a few limitations to the cohort of participants. For example, all pregnancies were singletons. The majority (90%) of participants were in the third trimester, thus the impacts of PPGP may be different earlier in pregnancy. However, we know PPGP prevalence increases with advancing gestation [6], hence the reported themes will be common to most women experiencing pain. All the participants were married or in de-facto relationships, hence the findings may have been different if single women were included as they may report different social support(s) or a lack thereof. The use of focus groups may have added to the richness of the information, but were not able to be conducted due to the social distancing restrictions during the COVID-19 global pandemic. Therefore, it is not known if the information offered in a group setting would be different to the responses provided in a one-to-one interview.

### Implications for health care

The findings point to the complexity of living with PPGP as women struggle to complete household tasks, wrangle with feelings of guilt and frustration, whilst trying to meet societal expectations placed on them. The plight of women, as told in this study, highlights the need to improve attitudes and approaches in caring for pregnant women, both in the community and in the health care sector.

Public health approaches, such as education campaigns, may be required to promote the important role partners and families have in supporting pregnant women, and aid change in community attitudes towards women, enabling them to seek assistance without fear of judgement. Government policies may also need to be examined, such as flexible working arrangements and childcare rebates, to ensure adequate social support is provided in pregnancy. It is essential that health care services provide timely education, endorse self-care approaches, and adopt patient-focused models of care, including expert assessment and referral pathways, to ensure all women have access to the right care, by the right person, at the right time.

## Conclusion

The unexpected reality of pain and associated limitations in physical capability, compounded by feelings of judgement from others, creates a need for timely intervention and resources for women with PPGP. Women expressed that this was not how they had envisaged their pregnancies would be and experienced a disruption that dramatically transformed their daily lives and social interactions. Women rely on social supports to cope with the emotional and functional impacts of PPGP on daily life, and advocate for a self-care approach to maintain a positive mindset and reduce stress. The findings suggest women seek more from health care professionals, including early education, personalised information, and prompt referrals for treatment, to assist in the management of PPGP. There is a clear need for the provision of empathetic community support and responsive healthcare services to support women with pain to better cope during pregnancy and improve their experiences at this critical time of their lives.

## Data Availability

All data generated or analysed during the current study are included in this published article.

## Abbreviations

PPGP: Pregnancy-related pelvic girdle pain
COREQ: Consolidated criteria for reporting qualitative research
COVID-19: Coronavirus disease of 2019
SD: Standard deviation
